# Analysis of Avian Orthoavulavirus 1 Detected in the Russian Federation between 2017 and 2021

**DOI:** 10.3390/vaccines11061032

**Published:** 2023-05-27

**Authors:** Nelly A. Guseva, Sergey N. Kolosov, Nikolay G. Zinyakov, Artem V. Andriyasov, Renfu Yin, Lidya O. Scherbakova, Evgenia V. Ovchinnikova, Zoya B. Nikonova, Dmitry B. Andreychuk, Alexander V. Sprygin, Ilya A. Chvala, Natalia V. Moroz

**Affiliations:** 1 Reference Laboratory for Avian Viral Diseases, FGBI “Federal Centre for Animal Health” (FGBI “ARRIAH”), 600901 Vladimir, Russia; 2 Key Laboratory of Zoonosis Research, Ministry of Education, College of Veterinary Medicine, Jilin University, Changchun 130062, China

**Keywords:** *AOAV-1*, phylogenetic analysis, Newcastle disease virus, fusion (F) gene, genotype

## Abstract

Newcastle disease virus (*NDV, Avian orthoavulavirus type 1, AOAV-1*) is a contagious high-impact poultry pathogen with infections detected worldwide. In the present study, 19,500 clinical samples from wild bird species and poultry collected from 28 regions of Russia between 2017 and 2021 were screened for the presence of the *AOAV-1* genome. NDV RNA was detected in 15 samples from wild birds and 63 samples from poultry. All isolates were screened for a partial sequence of the fusion (F) gene that included the cleavage site. Phylogenetic analysis demonstrated that lentogenic *AOAV-1* I.1.1, I.1.2.1, and II genotypes were dominant among vaccine-like viruses in the territory of the Russian Federation. A vaccine-like virus with a mutated cleavage site (112-RKQGR^L-117) was detected in turkeys. Among the virulent *AOAV-1* strains, viruses of the XXI.1.1, VII.1.1, and VII.2 genotypes were identified. The cleavage site of viruses of the XXI.1.1 genotype had a *112-KRQKR^F-117 amino acid sequence*. The cleavage site of viruses with VII.1.1 and VII.2 genotypes had a *112-RRQKR^F-117 amino acid sequence*. The data collected by the present study demonstrate the distribution and dominance of the virulent VII.1.1 genotype in the Russian Federation between 2017 and 2021.

## 1. Introduction

Newcastle disease (ND) is an annually reported viral disease in poultry, including ducks, pigeons, geese, and turkeys. ND is of considerable economic significance for the poultry industry due to its impact and is reportable to the World Organization for Animal Health. Newcastle disease is caused by virulent strains of *Avian orthoavulavirus type 1* (*AOAV-1*), also known as Newcastle disease virus (NDV), of the genus *Orthoavulavirus* belonging to the family *Paramyxoviridae* (International Committee on Taxonomy of Viruses). *AOAV-1* has wide genetic diversity. All *AOAV-1* strains and isolates are currently subdivided into two classes: class I and class II. Class I comprises genotype 1 only, which is subdivided into sub-genotypes 1.1.1, 1.1.2, and 1.2. Class II comprises at least 20 different genotypes, some of which are subdivided into sub-genotypes [1]. Recent studies have analyzed the NDV genome, including the F gene, to delineate the molecular evolution of circulating ND strains [2,3,4]. Cleavage of the F gene protein is considered to be a major determinant of *AOAV-1* virulence. Following replication, the fusion gene is translated into a precursor protein, F0, followed by cleavage by host cell proteases into F1 and F2 subunits to produce infectious viral particles. The amino acid sequence at the F protein cleavage site is used to classify *AOAV-1* strains according to low virulence (lentogenic pathotype) or high virulence (NDV or velogenic pathotype) [5,6,7].

Timely identification of circulating mesogenic and velogenic strains plays a key role in maintaining disease-free status, which can be accomplished by phylogenetic studies. Russia is crossed by many major migration flyways and serves as the major breeding area for many migratory species in the Palearctic. Identifying wildlife reservoirs may help predict new epizootics and facilitate the implementation of preventative measures. The most recent extensive studies of NDV in the territory of the Russian Federation and neighboring countries were conducted in 2010 [8]. The F gene fragment was comparatively analyzed in 79 NDV strains isolated from domestic and synanthropic birds in Kazakhstan, Kirghizia, Ukraine, and Russia between 1993 and 2007. All newly characterized isolates belonged to three NDV genotype VII subgroups: VIIa, VIIb, and VIId. Since then, no comparable studies have been undertaken despite the ongoing reports of NDV cases.

The current epidemiological situation shows the need to monitor NDV since the spread of NDV among migratory and synanthropic birds (pigeons, crows, and jackdaws) pose a serious threat to the commercial poultry industry. The goal of this study is to carry out a phylogenetic analysis of nucleotide sequences of the F gene of *AOAV-1* isolates from avian species from the wild and poultry in an area stretching all over Russia in 2017–2021.

## 2. Materials and Methods

### 2.1. Samples

Samples of avian biological material (internal organs, feces, and cloacal or tracheal swabs) were used in the present study. Samples were collected by inspectors of the Federal Service for Veterinary and Phytosanitary Surveillance and Veterinary Departments from poultry on commercial farms or in backyards and from wild and synanthropic birds. A total of 19,500 samples were accessioned for the present study.

### 2.2. RNA Extraction

RNA was extracted using RNeasy Mini Kit (Qiagen, Hilden, Germany) according to the manufacturer’s instructions.

### 2.3. PCR Assays

For diagnostic purposes, M gene-targeted real-time RT-PCR was performed with a Qiagen OneStep RT-PCR Kit (Qiagen, Hilden, Germany, forward primer (M4100F, 5′-agt-gat-gtg-ctc-gga-cct-tc-3′), reverse primer (M4220R, 5′-atc-gtt-tac-gga-gag-gag-tcc-3′), and probe (M4169, 5′-ttc-tct-agc-agt-ggg-aca-gcc-tgc-3′) using Rotor Gene Q machines (Qiagen, Hilden, Germany) [9]. RT-PCR was performed under the following temperature and time conditions: reverse transcription, 30 min at 50 °C; polymerase activation, 10 min at 95 °C, and 40 cycles of denaturation (10 s at 95 °C), annealing of primers (35 s at 55 °C), and elongation (10 s at 72 °C).

F gene-targeted RT and conventional PCR were performed using AMV reverse transcriptase and GoTaq^®^Flexi DNA polymerase (Promega, Madison, USA), forward primer (D1, 5′-cca-ttg-atg-gca-ggc-ctc-tt-3′), and reverse primer (D2, 5′-ccg-cta-ccg-att-aat-gag-ct-3′) following the manufacturer’s instructions. The reaction was performed using a Tercyc thermal cycler (DNA-Technology, Moscow, Russia) under the following temperature and time conditions: reverse transcription (25 min at 50 °C), polymerase activation (5 min at 95 °C), and 40 PCR cycles including denaturation (20 s at 95 °C), annealing of primers (30 sec at 55 °C), and elongation (40 s at 72 °C).

### 2.4. Sequencing

Nucleotide sequences of *AOAV-1* F and HN genes were determined using an automated ABI Prism 3100 sequencer and BigDye Terminator Cycle Sequencing kits (Applied Biosystems, Waltham, MA, USA) according to the manufacturer’s instructions. Nucleotide sequences were analyzed using BioEdit software version 7.0.5.3. Sequences were aligned using ClustalW multiple sequence alignment software. Phylogenetic trees were constructed according to the NJ algorithm using MEGA software version 6.06. The sequences presented in this study have been added to the database “GenBank” under the numbers OQ435162–OQ435239 and OQ473027-OQ473028.

### 2.5. The Geographical Map

The Geographical Map Was Obtained from the Map Chart Service (https://www.mapchart.net/index.html, accessed on 10 May 2023).

## 3. Results

Between 2017 and 2021, 7410 samples of biological material from wild and synanthropic birds collected from four regions of Russia and 12,090 samples of biological material collected from poultry on commercial poultry farms or in backyards from twenty-eight regions were tested for *AOAV-1* at the Reference Laboratory for Avian Viral Diseases in the FGBI “ARRIAH” (Vladimir, Russia). The species and groups of birds from which samples of biological material were obtained are presented in Table 1.

The greatest number of samples of biological material from wild and synanthropic birds was obtained from species of the order *Anseriformes* (4066 samples). This group predominantly comprised samples obtained from the family *Anatidae*, which includes ducks, mallards, wild geese, and teals. Samples from other wild waterfowl and near-water birds (gulls, grebes, and cormorants among others) were also examined (1588 samples). *AOAV-1* was detected in six samples from mallards (collected in 2017 or 2021), in one sample from gadwall (collected in 2017), in two samples from wild duck (collected in 2018), and in one sample from wild waterfowl (collected in 2020, species unknown).

A total of 1071 samples of biological material were received from synanthropic birds between 2017 and 2021. The greatest number of samples were obtained from rock pigeons (839 samples), with 232 samples obtained from crows, jackdaws, and sparrows, and 685 samples from other wild bird species (woodcock, lapwing, and pheasant among others). The *AOAV-1* genome was detected in five samples from pigeons in 2017, 2018, and 2020.

The greatest number of samples from poultry were obtained from chickens (9580 samples), with 2510 samples from turkeys, quails, domestic geese, ducks, and other domestic birds (guinea fowl and Muscovy duck among others). The *AOAV-1* genome was detected in fifty-eight samples from chickens (collected between 2017 and 2021), in two samples from turkeys (collected in 2018), in one sample from domestic geese (collected in 2019), and in two samples from poultry (collected in 2019 and 2020, species unknown).

The virus was detected in 78 samples collected from poultry and wild birds. Data on *AOAV-1* isolates are provided in Table 2.

**Figure 1:**
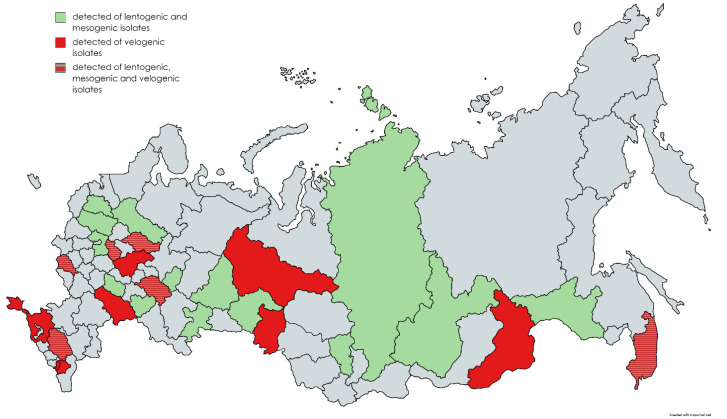
Regions of the Russian Federation where the *AOAV-1* was detected of between 2017 and 2021.

It should be noted that it was not possible to detect *AOAV-1* for a number of years in a number of regions of the Russian Federation. At the same time, various *AOAV-1* strains were repeatedly detected over a five-year period in much smaller geographic locations (Republic of Tatarstan, Vladimir, Kostroma, and Kursk regions). The regions of the Russian Federation where *AOAV-1* was detected are shown in Figure 1. 

**Figure 2:**
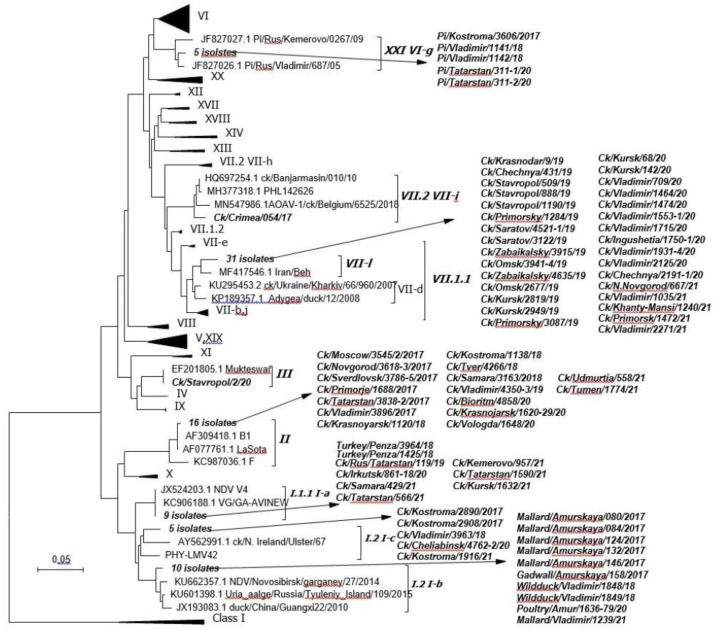
Phylogenetic positions of *AOAV-1* isolates recovered in the Russian Federation between 2017 and 2021. The tree was constructed using the NJ method with MEGA 6.06 software using F gene fragment sequences (nucleotides 203–550).

The virulent *AOAV-1* genotype XXI.1.1 was isolated from pigeons in the Kostroma and Vladimir Oblasts between 2017 and 2018 and in the Republic of Tatarstan in 2020 (five isolates; Figure 2). The cleavage site of viruses of genotype XXI.1.1 had an amino acid sequence of *112-KRQKR^F-117*.

Avirulent genotype I.2 viruses were isolated from wild waterfowl in the Amur and Vladimir Oblasts between 2017 and 2018 and between 2020 and 2021 (ten isolates). The cleavage site of viruses of genotype I.2 had an amino acid structure of *112-GKQGR^L-117*.

Virulent isolates of genotype VII.2 were recovered from domestic poultry in the Republic of Crimea in 2017 (one isolate). The cleavage site of viruses of genotype VII.2 had an amino acid structure of *112-RRQKR^F-117*.

The collected data demonstrate the widespread distribution and dominance of the virulent genotype VII.1.1. in the Russian Federation between 2019 and 2021. The virulent *AOAV-1* genotype VII.1.1 was detected in Krasnodar, Stavropol, Primorsky and Zabaikalsky Krais, the Republics of Chechnya and Ingushetia, Saratov, Omsk, Kursk, Vladimir and Nizhny Novgorod Oblasts, and Khanty-Mansi AO (31 isolates). The cleavage site of viruses of genotype VII.1.1 had an amino acid sequence of *112-RRQKR^F-117*.

The phylogenetic positions of *AOAV-1* isolates detected in the Russian Federation between 2017 and 2021 are shown in Figure 2.

**Figure 3:**
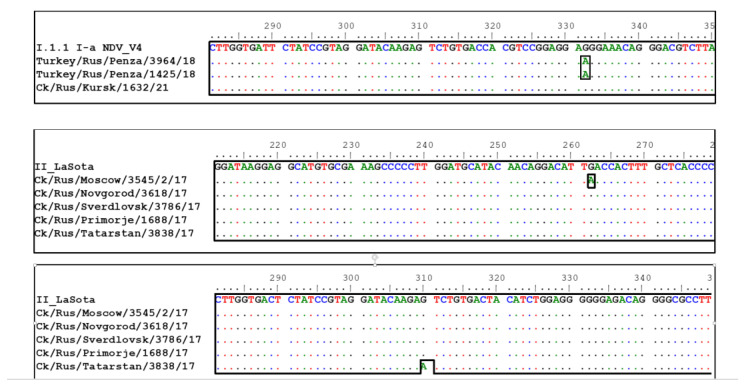
Nucleotide substitutions in the studied region of the gene F in *AOAV-1* vaccine-like viruses (nucleotides 203–550).

Vaccine-like virus genotypes I.1.1 and I.1.2.1 were detected in chickens and turkey in Penza, Irkutsk, Samara, Kemerovo, Kursk, Kostroma, Vladimir, and Chelyabinsk Oblasts and in the Republic of Tatarstan between 2017 and 2021 (14 isolates). Genotype II vaccine-like viruses were detected in chickens in Moscow, Novgorod, Sverdlovsk, Vladimir, Kostroma, Tver, Samara, Vologda, and Tyumen Oblasts, Primorsky and Krasnoyarsk Krais, and in the Republic of Tatarstan and Udmurt Republic between 2017 and 2021 (16 isolates). Genotype III vaccine-like virus was isolated from chickens in the Stavropol Krai in 2020 (one isolate). In our studies, only a few vaccine-like viruses were identified that had nucleotide substitutions in the studied region of the F gene (Figure 3).

Of particular interest is the substitution in the turkey/Rus/Penza/3964/18 virus at position 334 of the ORF of gene F. The substitution of G for A leads to the appearance of the basic amino acid R in the cleavage site. As a result, the cleavage site in the isolate has an amino acid sequence of 112-RKQGR^L-117, while the cleavage site in the vaccine strain V4 has an amino acid sequence of 112-GKQGR^L-117. This substitution was again detected from the same poultry farm turkey/Rus/Penza/1425/18 four months later (Table 2).

Analysis of the HN gene of the turkey/Rus/Penza/3964/18 and turkey/Rus/Penza/1425/18 viruses also demonstrated a significant relationship with the V4 vaccine. In the studied region of the HN gene (nucleotides 70–430), one G to A substitution was noted at position 322 of the ORF of the HN gene in both viruses. In the turkey/Rus/Penza/1425/18 virus, a substitution of T for C was also detected at position 342 of the ORF of the HN gene.

## 4. Discussion

Vaccination of commercial poultry against Newcastle disease, which is mandatory in the Russian Federation, is responsible for the regular detection of *AOAV-1* vaccine-like viruses. The majority of ND vaccines are lentogenic class II *AOAV-1* strains of genotypes I (strains V4 and BOR74 of sub-genotypes I.1.1 and I.1.2.1, respectively) and II (strains LaSota and B1), while a few are class II mesogenic strains of genotype II (Roakin, Komarov, and Beaudette C) and III (H). Lentogenic isolates of sub-genotypes I.1.1 and I.1.2.1 are also detected in wild birds [11]. Regarding the genotype II vaccine strains, LaSota and B1 strains are prevalent in Russia. Genotype II isolates were first reported in North America and were further detected in Africa, Asia, Europe, and South America [12,13,14,15]. The viruses of this genotype are currently used as live vaccines for backyard and commercial poultry in different regions of the world [15].

Long-term use of live vaccines probably leads to natural variability in isolates from poultry farms and the appearance of substitutions in manufacturing factories [16,17]. It is not always possible to determine whether isolates are a vaccine or a circulating vaccine-like isolate based on sequencing results. In our studies, only a few vaccine-like viruses were identified that had nucleotide substitutions in the examined fragment of the F gene. Examination of this short nucleotide sequence does not always distinguish between vaccines and vaccine-like viruses that may have re-infected birds. The repeated detection of a vaccine-like virus from a material in the Penza region indicates the possibility of vaccine strains acquiring pathogenic properties. The possibility of *AOAV-1* acquiring virulent properties has already been reported in Australia [18]. The appearance of an additional substitution at position 342 of the gene NH four months later for the virus turkey/Rus/Penza/1425/18 indicates the circulation of a vaccine-like virus among susceptible livestock and further evolution of this strain.

In 2020, the vaccine strain of class II genotype III was detected in backyard chickens in Stavropol Krai. The available data on genotype III isolate sequences suggest that all viruses of this genotype have virulent properties [15]. This genotype includes related strains H and Mukteswar, which are mesogenic and used as vaccines in some regions of the world [19,20].

In 2017, 2018, 2020, and 2021, class II genotype I.2 *AOAV-1* isolates were recovered in the Russian Federation. All isolates were detected in wild waterfowl. This subgenotype comprises avirulent viruses isolated in many European, Asian, African, and North American countries from different wild and domestic waterfowl species as natural reservoirs [15]. A number of such isolates have also been detected in Russia [21].

*AOAV-1* isolates of genotype VIg (according to the classification of Diel 2012 et al.) [10] or genotype XXI.1.1 (according to the classification of Dimitrov 2019 et al.) [1] are repeatedly reported in the Russian Federation. Genotype VI viruses, sometimes called pigeon paramyxovirus type 1 or PPMV-1, are considered panzootic. The majority of the genotype VI virus sub-genotypes have predominantly been isolated from birds of the Columbidae family, which includes different species of wild and domestic pigeons. The viruses of this genotype appear to have spread worldwide due to the trade in sport pigeons and natural migration of wild pigeons [15,22]. Various genotype VI *AOAV-1* variants and their derivatives are continuously circulating in Russia [22,23,24].

Between 2017 and 2021, NDV isolates of class II genotype VII were sporadically detected in poultry in Russia. In 2017, NDV isolates of subgenotype VII.2.1 were detected in the Republic of Crimea. This subgenotype was reported in Indonesia, Israel, and Pakistan between 2010 and 2013, and it further spread over Eastern Europe [25]. Previously, viruses of subgenotype VII.2.2, closely related to subgenotype VII.2.1, were detected in the Kaliningrad region of the Russian Federation in 2013. The virus was detected in backyard chickens [26]. This subgenotype, also related to viruses previously circulating in Indonesia (1998–2010), was also reported in poultry in Malaysia (2004–2006), China (2011, 2012), and Cambodia (2012). The vast and rapid spread of these genotypes in poultry indicates the high panzootic potential of this group of viruses. In addition, these groups of viruses are unlikely to have originated from isolates previously identified from poultry but rather from strains isolated from wild birds in the 1980s [27]. However, viruses of this genotype have not generally been widely distributed in the Russian Federation.

In 2019, a genotype VII.1.1 NDV isolate was detected in the Krasnodar Krai, and was responsible for a large-scale ND outbreak in between 2019 and 2021. Phylogenetic analysis of these nucleotide sequences demonstrated that the group of Iranian isolates designated as VII.1.1 (VIIL) was the most closely related to the Russian isolates from 2019–2021 (Figure 2) [28,29,30]. The rapid spread of the NDV genotype VII.1.1 across the territory of the Russian Federation in 2019 indicates that birds are the main vector for the spread of the ND virus. The low detection rate of NDV in wild birds in the present study contradicts this hypothesis. Despite the rare detection of NDV from wild birds, cases of genotype VII.1.1 NDV detected in wild birds have been reported. NDV was detected in a wild bird in Turkey [31], from two waterfowl (Common Moorhen and Mallard), in tissue samples taken from two little owls (Little_Owl/1/Istanbul/TR/2018 and Little_owl/2/Istanbul/TR/2018), and in one common kestrel (Common_kestrel/Istanbul/TR/2018). The NDV genotype VII.1.1 was detected in cattle egrets and house sparrows by RRT-PCR in Egypt during outbreaks between 2017 and 2019 [32]. However, a small proportion of previous studies have reported the detection of virulent viruses. According to the results of previous virus isolation studies, the prevalence of *AOAV-1* prevailed is between 0.5% and 2.5% in waterfowl, including ducks [33,34]. In our opinion, it is worth highlighting the main hypothesis for such a massive spread of the NDV genotype VII.1.1 across the territory of the Russian Federation: the spread of NDV only by certain species of birds. A small number of bird species act as carriers of NDV, which is confirmed by the absence of mass epizootics during the migration of infected birds, in contrast to the spread of the avian influenza virus where mass deaths are recorded among susceptible species. This limited number of bird species should include synanthropic birds. According to the results of monitoring studies conducted in Kazakhstan and covering 73 species of birds, virulent viruses were detected only in pigeons (Columbiformes) and a velogenic NDV of genotype XIII was also detected in cormorants [35]. The results of a recent study of ND outbreaks in Kazakhstan indicate that NDV outbreaks of genotype VIIb (according to the classification of Diel et al.) in Central Asia are associated with cormorant migration [36]. These studies confirm the role of wild and synanthropic birds in the spread of *AOAV-1* in poultry. At the same time, numerous vaccine-like viruses have been detected in wild and synanthropic birds, including Peregrine falcon/Brazil/PET26711/2009 (KU133356.1), River Eagle/Nigeria/PL JZ04/N49/896/2002–2003 (MH996916.1), Vulture/Nigeria/PL038-II/N47/895/2002–2003 (MH996914.1), Duck/Pakistan/ISLM/AW-17/2015 (MG686586.1), Mute swan/Bulgaria/Malko Tarnovo/2006 (KU133353.1), Eurasian_Teal/China/Ningxia/6/2014 (KT282108.1), and Pigeon/China/Heilongjiang/YA05/2014 (KU200240.1) et al.), which may indicate immunity to the *AOAV-1* among synanthropic birds. Apparently, constant contact with vaccine-like and avirulent viruses protects synanthropic birds from mass death upon contact with velogenic NDV. Accordingly, the results of the present study demonstrate the active circulation of various *AOAV-1* strains in the territory of the Russian Federation and the need for enhanced surveillance of synanthropic birds.

## Figures and Tables

**Table 1:** Species and groups of birds from which samples were obtained.

Bird Species	2017	2018	2019	2020	2021
Poultry					
Chicken	858	1018	2295	2484	2925
Turkey	172	55	258	72	77
Quail	-	-		-	41
Domestic geese and ducks	59	144		171	139
Other domestic birds (guinea fowl, Muscovy duck, etc.)	281		432	323	286
Total samples from poultry	1370	1217	2985	3050	3468
Wild waterfowl					
Wild duck	300	278	515	358	218
Mallard	214	90	259	76	183
Wild geese	237	87	173	247	179
Teal	45	-	93	59	143
Others of the family Anatidae	59	20	58	28	147
Other wild waterfowl and near-water birds (gulls, grebes, cormorants, etc.)	57	167	433	587	344
Synanthropic birds and other wild bird species					
Rock pigeon	102	39	177	343	178
Others of the order Passerine (crows, jackdaws, and sparrows)	13	35	95	60	29
Woodcock	40	19	102	38	26
Other wild bird species (lapwing, pheasant, etc.)	63	48	110	154	85
Total samples from synanthropic and wild birds	1130	783	2015	1950	1532
Total samples per year	2500	2000	5000	5000	5000

**Table 2:** *AOAV-1* isolates recovered in the Russian Federation between 2017 and 2021.

No.	Region	Date of Sample Collection	Species	Isolate Designation
Genotype I.1.1 (Ia, V4) ^a^				
1	Penza Oblast	07.02.18	Turkey	turkey/Rus/Penza/3964/18
2	Penza Oblast	23.06.18	Turkey	turkey/Rus/Penza/1425/18
3	Republic of Tatarstan	09.04.19	Chicken	chicken/Rus/Tatarstan/119/19
4	Irkutsk Oblast	23.06.20	Chicken	chicken/Rus/Irkutsk/861/20
5	Samara Oblast	08.04.21	Chicken	chicken/Rus/Samara/429/21
6	Republic of Tatarstan	29.04.21	Chicken	chicken/Rus/Tatarstan/566/21
7	Kemerovo Oblast	05.07.21	Chicken	chicken/Rus/Kemerovo/957/21
8	Republic of Tatarstan	07.10.21	Chicken	chicken/Rus/Tatarstan/1590/21
9	Kursk Oblast	13.10.21	Chicken	chicken/Rus/Kursk/1632/21
Genotype I.2 (Ib) ^a^				
1	Amur Oblast	23.11.17	Mallard	mallard/Rus/Amur/80/17
2	Amur Oblast	23.11.17	Mallard	mallard/Rus/Amur/84/17
3	Amur Oblast	23.11.17	Mallard	mallard/Rus/Amur/124/17
4	Amur Oblast	23.11.17	Mallard	mallard/Rus/Amur/132/17
5	Amur Oblast	23.11.17	Mallard	mallard/Rus/Amur/146/17
6	Amur Oblast	23.11.17	Gadwall	gadwall/Rus/Amur/158/17
7	Vladimir Oblast	12.07.18	Wild duck	wildduck/Rus/Vladimir/1848/18
8	Vladimir Oblast	12.07.18	Wild duck	wildduck/Rus/Vladimir/1849/18
9	Amur Oblast	02.10.20	Wild waterfowl	wildwaterfowl/Rus/Amur/1636/20
10	Vladimir Oblast	23.08.21	Mallard	Mallard/Rus/Vladimir/1239/21
Genotype I.1.2.1 (Ic; BOR74) ^a^				
1	Kostroma Oblast	19.09.17	Chicken	chicken/Rus/Kostroma/2890/17
2	Kostroma Oblast	19.09.17	Chicken	chicken/Rus/Kostroma/2908/17
3	Vladimir Oblast	07.02.18	Chicken	chicken/Rus/Vladimir/3963/18
4	Chelyabinsk Oblast	17.03.20	Chicken	chicken/Rus/Cheliabinsk/4762/20
5	Kostroma Oblast	18.11.21	Chicken	chicken/Rus/Kostroma/1916/21
Genotype II (La-Sota, B1) ^a^				
1	Moscow Oblast	21.01.17	Chicken	chicken/Rus/Moscow/3545/2/17
2	Novgorod Oblast	04.04.17	Chicken	chicken/Rus/Novgorod/3618/17
3	Sverdlovsk Oblast	30.06.17	Chicken	chicken/Rus/Sverdlovsk/3786/17
4	Primorsky Krai	18.07.17	Chicken	chicken/Rus/Primorje/1688/17
5	Republic of Tatarstan	30.08.17	Chicken	chicken/Rus/Tatarstan/3838/17
6	Vladimir Oblast	01.11.17	Chicken	chicken/Rus/Vladimir/3896/17
7	Krasnoyarsk Krai	23.05.18	Chicken	chicken/Rus/Krasnoyarsk/1120/18
8	Kostroma Oblast	24.05.18	Chicken	chicken/Rus/Kostroma/1138/18
9	Tver Oblast	16.10.18	Chicken	chicken/Rus/Tver/4266/18
10	Samara Oblast	18.12.18	Chicken	chicken/Rus/Samara/3163/2018
11	Vladimir Oblast	17.01.19	Chicken	chicken/Rus/Vladimir/4350/19
12	Vladimir Oblast	15.05.20	Chicken	chicken/Rus/Bioritm/4858/20
13	Krasnoyarsk Krai	10.11.20	Chicken	chicken/Rus/Krasnojarsk/1620/20
14	Vologda Oblast	23.11.20	Chicken	chicken/Rus/Vologda/1648/20
15	Udmurt Republic	29.04.21	Chicken	chicken/Rus/Udmurtia/558/21
16	Tyumen Oblast	01.11.21	Chicken	chicken/Rus/Tumen/1774/21
Genotype III (H) ^a^				
1	Stavropol Krai	11.12.20	Chicken	chicken/Rus/Stavropol/2/20
Genotype XXI.1.1 (VIg) ^a^				
1	Kostroma Oblast	28.03.17	Pigeon	pigeon/Rus/Kostroma/3606/17
2	Vladimir Oblast	14.06.18	Pigeon	pigeon/Rus/Vladimir/1141/18
3	Vladimir Oblast	14.06.18	Pigeon	pigeon/Rus/Vladimir/1142/18
4	Republic of Tatarstan	13.03.20	Domestic pigeon	pigeon/Rus/Tatarstan/311-1/20
5	Republic of Tatarstan	13.03.20	Domestic pigeon	pigeon/Rus/Tatarstan/311-2/20
Genotype VII.2.1 (VIIi) ^a^				
1	Republic of Crimea	12.01.17	Chicken	chicken/Rus/Crimea/054/17
Genotype VII.1.1 (VIIL) ^a^				
1	Krasnodar Krai	01.02.19	Chicken	chicken/Rus/Krasnodar/11/19
2	Republic of Chechnya	12.04.19	Chicken	chicken/Rus/Chechnya/431/19
3	Stavropol Krai	23.04.19	Chicken	chicken/Rus/Stavropol/509/19
4	Stavropol Krai	26.04.19	Chicken	chicken/Rus/Stavropol/888/19
5	Stavropol Krai	15.05.19	Goose	goose/Rus/Stavropol/1190/19
6	Primorsky Krai	22.05.19	Chicken	chicken/Rus/Primorsky/1284/19
7	Saratov Oblast	20.06.19	Chicken	chicken/Rus/Saratov/4521/19
8	Saratov Oblast	27.08.19	Chicken	chicken/Rus/Saratov/3122/19
9	Zabaikalsky Krai	04.10.19	Chicken	chicken/Rus/Zabaikalsky/3915/19
10	Omsk Oblast	10.10.19	Chicken	chicken/Rus/Omsk/3941/19
11	Zabaikalsky Krai	21.10.19	Chicken	chicken/Rus/Zabaikalsky/4635/19
12	Omsk Oblast	08.11.19	Chicken	chicken/Rus/Omsk/2677/19
13	Kursk Oblast	18.11.19	Poultry	poultry/Rus/Kursk/2819/19
14	Kursk Oblast	03.12.19	Chicken	chicken/Rus/Kursk/2949/19
15	Primorsky Krai	19.12.19	Chicken	chicken/Rus/Primorsky/3087/19
16	Kursk Oblast	20.01.20	Chicken	chicken/Rus/Kursk/68/20
17	Kursk Oblast	30.01.20	Chicken	chicken/Rus/Kursk/142/20
18	Vladimir Oblast	15.05.20	Chicken	chicken/Rus/Vladimir/709/20
19	Vladimir Oblast	09.09.20	Poultry	poultry/Rus/Vladimir/1464/20
20	Vladimir Oblast	09.09.20	Chicken	chicken/Rus/Vladimir/1474/20
21	Vladimir Oblast	17.09.20	Chicken	chicken/Rus/Vladimir/1553/20
22	Vladimir Oblast	13.10.20	Chicken	chicken/Rus/Vladimir/1715/20
23	Republic of Ingushetia	13.10.20	Chicken	chicken/Rus/Ingushetia/1750/20
24	Vladimir Oblast	10.11.20	Chicken	chicken/Rus/Vladimir/1931/20
25	Vladimir Oblast	02.12.20	Chicken	chicken/Rus/Vladimir/2125/20
26	Republic of Chechnya	11.12.20	Chicken	chicken/Rus/Chechnya/2191/20
27	Nizhny Novgorod Oblast	18.05.21	Chicken	chicken/Rus/N.Novgorod/667/21
28	Vladimir Oblast	20.07.21	Chicken	chicken/Rus/Vladimir/1035/21
29	Khanty-Mansi AO	23.08.21	Chicken	chicken/Rus/Khanty-Mansi/1240/21
30	Primorsky Krai	27.09.21	Chicken	chicken/Rus/Primorsk/1472/21
31	Vladimir Oblast	29.12.21	Chicken	chicken/Rus/Vladimir/2271/21

^a^ Phylogenetic groups are according to Dimitrov et al. (2019) and Diel et al. (2012) [1,10]. The strain designations of vaccines are indicated in brackets.

## Data Availability

The article shows the numbers under which the data was deposited in GenBank.
